# Classical V600E and other non-hotspot *BRAF* mutations in adult differentiated thyroid cancer

**DOI:** 10.1186/s12967-016-0958-x

**Published:** 2016-07-07

**Authors:** Avaniyapuram Kannan Murugan, Ebtesam Qasem, Hindi Al-Hindi, Yufei Shi, Ali S. Alzahrani

**Affiliations:** Molecular Endocrinology Section, Department of Molecular Oncology, King Faisal Specialist Hospital and Research Center, Research Center (MBC 03), PO Box 3354, Riyadh, 11211 Saudi Arabia; Department of Pathology and Laboratory Medicine, King Faisal Specialist Hospital and Research Center, Riyadh, Saudi Arabia; Department of Genetics, King Faisal Specialist Hospital and Research Center, Riyadh, Saudi Arabia; Department of Medicine, King Faisal Specialist Hospital and Research Center, Riyadh, Saudi Arabia

**Keywords:** Mutation, *BRAF*, Oncogene, DTC, Thyroid cancer, PTC, Saudi Arabia

## Abstract

**Background:**

*BRAF* is the most frequently mutated gene in differentiated thyroid cancer (DTC). Previous studies on DTC have well documented high rates of the *BRAF*^*V600E*^ mutation in patients of mixed ages. Previous studies either included a mix of pediatric and adult patients or pediatric patients only. However, the prevalence of hotspot and non-hotspot *BRAF* mutations and its significance in pure adult DTCs is not yet well determined. In this study we determine the frequency of this classical *BRAF* mutation and other rare *BRAF* mutations in pure adult DTCs.

**Methods:**

A total of 204 adult DTC samples (Age >18 years) were analyzed for mutations in exon 15 of the *BRAF* gene by performing polymerase chain reaction (PCR) amplification of tumor genomic DNAs and direct sequencing of amplicons using Sanger sequencing. Obtained results were correlated to clinical and pathological characteristics of DTCs. Statistical analyses were performed using SPSS (The Statistical Package for Social Sciences) version 20 software.

**Results:**

Overall, *BRAF* mutations were identified in 48.5 % (99/204) of adult DTCs. Three rare non-hotspot mutations (T599I, T599dup and K601E) were detected in four tumor samples (2 %). One (K601E) of these non-hotspot mutations occurred in conventional papillary thyroid cancer (CPTC) and other three (T599I, T599dup and K601E) were found in follicular variant PTC. We found significant association between *BRAF*^V600E^ mutation and age (*P* < 0.0001), extrathyroidal invasion (*P* = 0.017), lymph node metastasis (*P* = 0.038) and TNM stage III/IV (*P* = 0.001).

**Conclusions:**

Our study is the first to report *BRAF* mutations in a pure adult sample of DTCs of Saudi Arabian ethnicity. Our results show a high rate and a strong prognostic role of the classical *BRAF*^*V600E*^ mutation and also suggest a common occurrence of non-hot spot mutations in adult DTC from this highly inbred population.

## Background

Thyroid cancer is the most common malignant endocrine tumor. Its incidence has been considerably increasing over the last 4 decades [1, 2]. Thyroid cancer usually arises from follicular epithelial cell or parafollicular cells [3]. The latter is the cell of origin of medullary thyroid cancer [3]. Follicular cell-derived thyroid cancer is by far the most common type (95 %) and is classified to differentiated thyroid cancer (DTC), poorly differentiated (PDTC) and undifferentiated (anaplastic) subtypes [3]. DTC is further classified to papillary thyroid cancer (PTC) and follicular thyroid cancer (FTC) [28]. In Saudi Arabia, DTC is the fourth most common type of cancer in general and the second most common cancer in women [4]. Over the last decade, there has been a significant progress in our understanding of DTC pathogenesis [5]. Genetic alterations in genes of key cellular signaling pathways, particularly mitogen activated protein kinase (MAPK) and phosphatidylinositol-3-kinase (PI3K) signaling pathways are the main genetic mechanisms of thyroid carcinogenesis [5, 6].

PTC is the most common subtype of thyroid cancer accounting for about 90–95 % of cases. *BRAF*^V600E^ mutation is the most prevalent genetic alteration in PTC (~45 %) [5]. This transversion mutation results in thymine-to-adenine (T > A) change at nucleotide position 1799 (T1799A) substituting valine by glutamic acid at amino acid position 600 (V600E). When present, this mutation results in constitutive activation of the BRAF protein with consequent activation of the downstream mediators [5, 7, 8].

A large number of studies from different ethnic backgrounds reported *BRAF*^V600E^ mutation in 29–83 % of PTC [7]. These studies were mostly undertaken in patients with DTC of various ages. To our knowledge, no previous study reported *BRAF*^V600E^ mutation in a pure adult population. The relationship of *BRAF*^V600E^ mutation and age of the patient at diagnosis has been inconsistent. Some studies showed significant correlation [9, 10] while others didn’t demonstrate such a correlation [11]. We and others have found a much lower rate of *BRAF*^V600E^ mutation in DTC from pediatric and adolescent patients [12, 13]. Furthermore, we also found a much lower rate of the recently described *TERT* mutations in pediatric compared to adult DTC [13]. We speculated that the adult and pediatric DTCs are likely to have different molecular signatures as suggested by these findings and also the significantly different clinical and pathological phenotype [14–16]. Saudi population is highly inbred with high rate of consanguinity [17, 18]. Although *BRAF*^V600E^ is somatic in nature, one may wonder whether this high rate of consanguinity may have an impact on the prevalence of *BRAF*^V600E^ mutation and prevalence of other non-spot *BRAF* mutations. Although *BRAF*^V600E^ mutation has been previously reported from Saudi Arabian population of various ages [19, 20], it has not been investigated in purely adult population. In the course of this study, we also found a relatively high rate of non-hot spot *BRAF* mutations. Our aim in this study is to determine the prevalence of the *BRAF*^V600E^ mutation and other non-conventional exon 15 *BRAF* mutations in pure adult patients (>18 years) with DTC in this highly inbred population.

## Methods

### Tumor samples and DNA extraction

We studied a sample consisting of 204 sporadic DTCs. The clinical and pathological data of these patients are summarized in Table 1. The only exclusion criterion was age at diagnosis of ≤18 years. DTC samples included 114 (55.9 %) conventional papillary thyroid cancer (CPTC), 55 (27 %) follicular variant papillary thyroid cancer (FVPTC), 29 (14.2 %) tall cell papillary thyroid cancer (TC-PTC), 3 (1.5 %) hurthle cell carcinomas (HCC), and 3 (1.5 %) diffuse sclerosing type papillary thyroid cancer (DSC) collected from the pathology Department of the King Faisal Specialist Hospital and Research Centre (KFSH&RC), Riyadh, Saudi Arabia (Table 1). The patients did not have family history of thyroid cancer. Histological diagnosis was confirmed by an experienced endocrine pathologist (H.A) who also carefully selected the tumor samples from paraffin blocks to ensure that tumor tissue was dissected for DNA extraction. Slices of 10-micron thickness of tumor samples were dissected from formalin fixed and paraffin embedded tissue (FFPT). Genomic DNA was extracted from the tumor tissues as previously described [21] using the Gentra Puregene DNA extraction kit (Qiagen, Valencia, CA).

**Table 1:** Clinical and histopathological characteristic features of 204 cases of adult DTC

Characteristics	Number/total (percentage)
Median age in years (range)	35.5 (19–75)
Sex female:male (ratio)	154:50
Median tumor size in cm (range)	2.2 (1–12)
*Tumor sub-types (%)*	
Conventional papillary thyroid cancer (CPTC)	114 (55.9)
Follicular variant papillary thyroid cancer (FV-PTC)	55 (27)
Tall cell variant papillary thyroid cancer (TC-PTC)	29 (14.2)
Hurthle cell cancer (HCC)	3 (1.5)
Diffuse sclerosing type papillary thyroid cancer (DSC)	3 (1.5)
*Pathological characteristics (%)*	
Tumor multifocality	97 (47.5)
Extrathyroidal invasion	95 (46.6)
Lymph node metastasis	91 (44.6)
Distant metastasis	15 (7.4)
*TNM staging (%)*	
Stage I/II	160 (78.4)
Stage III/IV	44 (21.6)

### Ethical approval

This study was approved by the Ethical Committee and Institutional Review Board of King Faisal Specialist Hospital and Research Center, Riyadh, Saudi Arabia (RAC-2130015).

### PCR amplification and sequencing

Exon 15 of the *BRAF* gene was PCR amplified using genomic DNA from the tumor samples. The forward and reverse primers and the PCR conditions were as described previously [22]. We limited our search to exon 15 as the previously described mutations were mostly described in this exon. The PCR amplicons were confirmed on 2 % agarose gel and directly sequenced using the Big Dye terminator v3.1 cycle sequencing ready reaction kit (Applied Biosystems) and by ABI PRISM 3730X1, genetic analyzer (Applied Biosystems). Identified mutations were confirmed in both forward and reverse directions by an independent PCR amplification and sequencing reaction. For the confirmed mutations, matched normal tissue samples were further analyzed to check whether the mutations are somatic or germline in nature. The sequencing results were read against the *BRAF* gene (GeneBank No.: NM_004333.4).

### Statistical analysis

Statistical analyses were performed using SPSS (The Statistical Package for Social Sciences) version 20 software (IBM Corp., NY USA). Data were expressed as median and range for numerical values and numbers and percentages for categorical data. The T test was used to analyze continuous variables and Fisher exact and χ^2^ tests were used for categorical data. In all analyses, a two-tailed *P* < 0.05 was considered statistically significant.

### Availability of data and supporting materials

Paraffin embedded tumor tissues, genomic DNAs and data of tumor molecular genetics. The SPSS (The Statistical Package for Social Sciences) version 20 software was obtained from IBM Corp., NY USA. The protein molecules can be downloaded from http://www.rcsb.org/pdb/home/home.do using the PDB ID and can be viewed using SWISS PDB and Jmol viewer downloaded freely from http://www.spdbv.vital-it.ch/ and http://www.jmol.sourceforge.net/, respectively.

## Results

### Prevalence of BRAF mutation and identification of rare, non-hotspot BRAF mutation in adult DTCs

**Fig. 1:**
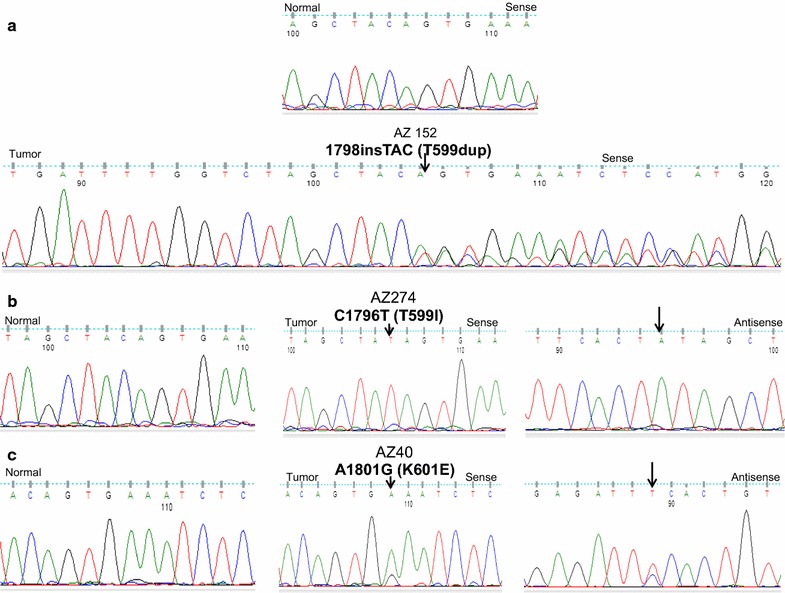
Identification of *BRAF* mutations in adult differentiated thyroid cancer. **a** The chromatogram shows a rare, somatic, non-hotspot, insertion mutation of *BRAF* gene in exon 15 at nucleotide position 1798 [1798insTAC (T599dup)]. **b** Shows a rare, somatic, non-hotspot, point mutation of *BRAF* gene in exon 15 at nucleotide position 1796 [C1796T (T599I)]. **c** A representative sequencing results shows a rare, somatic, non-hot spot, point mutation of *BRAF* gene in exon 15 at nucleotide position 1801 [A1801G (K601E)] from two independent tumor samples. In all the above cases, sequencing results of the matched normal tissue of each sample are shown in the left side of the panel. All the samples were repeated by independent PCR reactions with forward and reversed sequencing

Of 204 DTCs, 99 samples (48.5 %) harbored a *BRAF* mutation (Table 2). The hotspot *BRAF*^*V600E*^ mutation was found in 95 cases (46.5 %). As shown in Table 3, four samples pertaining to 4 unrelated patients harbored other non-hotspot *BRAF* mutations. One sample harbored C > T transition mutation at nucleotide position 1796 resulting in a T599I amino acid change. The tumor was a follicular variant papillary thyroid cancer (FV-PTC) from a female patient. The other sample harbored a very rare 3-base insertion mutation at nucleotide position 1798 resulting in duplication of threonine 599 (T599dup) and this mutation was identified in a tumor from a 44-year old lady with a 3-cm FV-PTC without extrathyroidal invasion, lymph node or distant metastases. Two samples, one in a 30-year old lady with CPTC without extrathyroidal invasion, lymph node or distant metastases and the other was in a 30-year old lady with 1 cm FV-PTC without extrathyroidal extension, lymph node or distant metastases, harbored an A > G point mutation at nucleotide position 1801 resulting in a K601E amino acid change (Fig. 1). These mutations were confirmed to be somatic in nature as they were absent in matched normal tissue samples. These three non-hot spot mutations have already been recorded in the COSMIC (Catalogue of Somatic Mutations in Cancer) database, UK.

**Table 2:** Summary of exon 15 *BRAF* mutations identified in 204 cases of adult DTC

Tumor subtype	BRAF^V600E^	*BRAF* ^K601E/T599I/T599dup^	Mutated/total sample (%)
CPTC	55	1	56/114 (49.1)
FV-PTC	13	3	16/55 (29)
TPTC	27	0	27/29 (93.1)
HCC	0	0	0/3 (0)
DSC	0	0	0/3 (0)
	*95/204 (46.5* *%)*	*4/204 (1.96* *%)*	*99/204 (48.5)*

**Table 3:** Various non-hotspot *BRAF* mutations identified in four out of 204 cases of adult DTC

Tumor no	Sex	Histology	Exon	Nucleotide	Codon	Amino acid	Mutation	Status
AZ 274	F	FV-PTC	15	C1796T	ACA-ATA	T599I	Missense	Heterozygous
AZ 152	F	FV-PTC	15	1798inTAC		T599dup	Insertion	In frame
AZ 40	F	CPTC	15	A1801G	AAA-GAA	K601E	Missense	Heterozygous
AZ 172	F	FV-PTC	15	A1801G	AAA-GAA	K601E	Missense	Heterozygous

*F* female; *FV*-*PTC* follicular variant papillary thyroid cancer; *CPTC* conventional papillary thyroid cancer

### Association of *BRAF*^V600E^ mutation with clinicopathological features and outcome of DTC

Patients included in this study were all adults and the mean ± SD age was 43.5 ± 15 years in patients with *BRAF*^*V600E*^ mutation and 36.2 ± 13.3 years in patients with wild type *BRAF* (*P* 0.0001). Extrathyroidal invasion was more frequently observed in tumors with *BRAF*^*V600E*^ mutation occurring in 54 tumors (56.8 %) compared to only 41 (39 %) tumors with wild type *BRAF* (*P* = 0.017). Lymph node metastasis was more commonly found in 51 tumors (53.7 %) with *BRAF*^*V600E*^ mutation compared to 40 tumors (38.1 %) with wild type *BRAF* (*P* = 0.038). Similarly, *BRAF*^V600E^ mutation was more common in higher stage tumors (stage III/IV) occurring in 31 patients (32.6 %) compared with only 13 (12.4 %) in the wild type *BRAF* (*P* = 0.001). *BRAF*^*V600E*^ mutation was not associated with gender, tumor size, tumor multifocality, distant-metastasis, and persistent/recurrent disease at 6–12 months after initial management and at the last follow up (Table 4).

**Table 4:** Comparison of clinical and histopathological characteristics between patients with positive and negative *BRAF* ^V600E^ mutation in adult DTCs

Characteristics	*BRAF* ^V600E^ mutation	*BRAF* wild type	*P* value
	(95 cases)	(105 cases)	
Age mean ± SD (years)	43.5 ± 15	36.2 ± 13.3	0.0001*
Sex (female:male)	71:24	79:26	1.00
Tumor size	3.05 ± 1.97	3.31 ± 2.43	0.42
Tumor multifocality	49 (51.6 %)	48 (45.7 %)	0.49
Extrathyroidal invasion	54 (56.8 %)	41 (39 %)	0.017*
Lymph node metastasis	51 (53.7 %)	40 (38.1 %)	0.038*
Distant metastasis	6 (6.3 %)	9 (8.6 %)	0.74
TNM stage III/IV	31 (32.6 %)	13 (12.4 %)	0.001*
Persistent disease (6–12 months after initial management)	47 (49.5 %)	43 (40.96 %)	0.29
Persistent/recurrent disease (At last follow up)	37 (38.9 %)	30 (28.6 %)	0.16

* Statistically significant

## Discussion

In this study, we analyzed exclusively a sample of adult DTC patients for *BRAF* mutations as it has been reported that adult DTCs harbor higher frequencies of *BRAF* mutations than pediatric DTC [13, 23, 24] and previous data showed significant differences in the clinical, pathological and molecular basis of pediatric compared with adult DTCs [15, 16, 25–28]. We report here 48.5 % (99/204) of *BRAF* mutations in adult DTC from Saudi Arabia. As expected, the vast majority of the identified mutations were the well described mutation, *BRAF*^V600E^ (95/204, 46.5 %). In addition, we also found three rare, non-hotspot *BRAF* mutations (T599I, T599dup and K601E) in four cases (4/204, 2 %). Although these non-hot spot mutations were previously described, their rate in this study seems higher than expected. Consanguinity is common in Saudi Arabia [17, 18] and although these mutations are somatic in nature, it is possible that there might be a genetic predisposition to their occurrence as inheritance of certain SNPs in the DNA repairing machinery genes may theoretically predispose to the development of *BRAF* and other driver mutations in PTC.

**Fig. 2:**
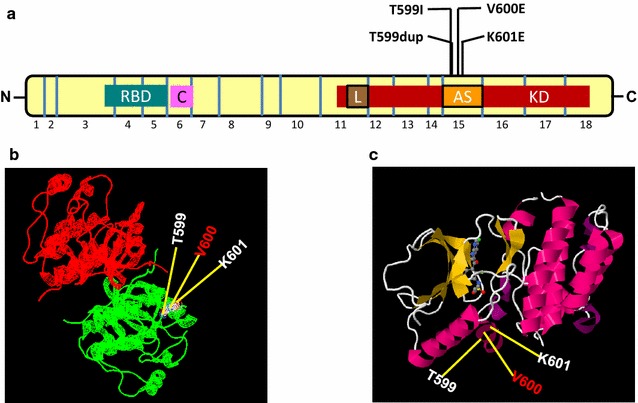
Schematic diagram of the *BRAF*. **a** Schematic diagram of the *BRAF* shows the rare, somatic, non-hot spot, mutations of *BRAF* gene in exon 15 at various nucleotide positions, numbers in the boxes indicate exons and boxes indicate various domains: RBD-ras binding domain, CRD-cysteine rich domain, KD-kinase domain, L–G loop, a conserved glycine motif. **b** A diagram of native modeled structure of BRAF homodimer. Each monomer molecule is indicated in different color (*red* and *green*). The structure of BRAF protein in complex PDB ID is 4E26. Amino acid residues of rare, non-hotspot, somatic mutation identified in adult differentiated thyroid cancer are plotted in BRAF native protein structure using SWISS PDB viewer and the mutated residues are shown in *sphere shape*. **c** Shows the monomer cartoon diagram of native modeled structure of BRAF kinase domain. The structure of BRAF protein kinase domain (monomer) PDB ID is 4WO5. Amino acid residues of rare, non-hotspot, somatic mutation identified in adult differentiated thyroid cancer are plotted in BRAF native protein structure and the molecule is visualized by JSmol

Detailed analyses of the previously reported *BRAF* mutations in PTCs showed that in addition to the most common *BRAF*^V600E^ mutation, many unique and rare non-hotspot mutations have been detected in the exon 15 of the *BRAF* gene and these mutations have recently been compiled in a report [29]. In our study, we found three rare, non-hotspot *BRAF* mutations in four cases. In a tumor from a 47-year old female with FV-PTC, we found the rare non-hotspot missense T599I mutation. This mutation has also been previously reported in a follicular variant [30] and in a solid variant [31] PTC. In both cases, the T599I mutation was originally identified as a concomitant mutation with a complex *BRAF* mutation V600delinsAL (replacement of valine with an alanine and a leucine) in the same allele, and VKSRdel (deletion of valine 600, lysine 601, serine 602, and arginine 603), respectively [30, 31]. However, we did not find any co-existing mutation with the T599I mutation in our patient’s tumor sample. Previously reported functional analysis of this mutation revealed that the T599I mutant had a moderately increased kinase activity but with dramatically increased phosphorylation and activation of ERK [8]. This phenomenon might be related to the effect of dimerization which is likely achieved via complexing with wild-type BRAF and RAF1 [32]. As illustrated in Fig. 2, this mutation is located in the core region of the kinase domain and more specifically in the activation loop that is encoded by the nucleotides of the *BRAF* exon 15. Moreover, the rare non-hotspot mutation T599I resides in one of the two critical phosphorylation sites (T599 and S602) in the activation loop. As BRAF is a serine/threonine kinase, it has been hypothesized that phosphorylation of Threonine 599 and Serine 602 residues disturb the hydrophobic interaction of P-loop (phosphate binding loop) with the A-loop (activation loop) [8]. Therefore, it is likely that phosphorylating either the S/T residues would substantially result in destabilization of inactive BRAF protein and constitutively switching to active conformation. In a previous report from Saudi Arabia, deletion of threonine at this position (T599del) was described in 1 out of 69 benign follicular adenomas, and 1 of 115 PTC harbored an insertion of additional threonine at this position (T599dup) [20]. This latter mutation was also detected in another patient from our series. This mutation was previously described in a pilocytic astrocytoma [33] and in anaplastic thyroid cancer (ATC) samples with tall cell like phenotypes [34, 35]. It has been demonstrated that the T599dup displays an increased kinase activity and enhanced MEK phosphorylation followed by ERK activation and potentially comparable to those of the hotspot *BRAF*^V600E^ mutation [33]. In addition, this insertion mutation has also been shown to transform the NIH3T3 and MCF-10A cells in culture. It has been speculated that insertion of an additional amino acid at this T599 residue of the protein backbone destabilizes the inactive conformation of the kinase domain which may result in its conversion to an active mutant BRAF [33].

We also identified another rare non-hotspot mutation (K601E) in two tumor samples of adult DTC, one from a CPTC and the other from a FV-PTC. This mutation has been well documented in DTC and is considered the second most common *BRAF* mutation in DTC after *BRAF*^V600E^. Furthermore, it also has been found to be associated with tumor cells with histological features of FVPTC [10], more specifically in the encapsulated follicular variant of PTC. Recently, it has been shown that this K601E mutation may also be found rarely in other sub-types of thyroid cancer including follicular thyroid carcinoma (FTC) [36]. In the study by Schulten et al. this mutation was found in three cases, an FTC, a PTC and a FV-PTC [20]. Transient transfection mediated functional characterization of this mutation in HEK 293T cells showed enhanced kinase activity resulting in subsequent strong MEK phosphorylation and activation of ERK1/2 kinases in the absence of TSH [30] suggesting that this is a typical gain-of-function mutation and likely to drive cell proliferation and thyroid carcinogenesis in a kinase dependent fashion. We found that all of these three rare non-hotspot mutations were in cases of FV-PTC and the K601E was also found in one case of CPTC. Interestingly, the histopathological features and the outcome of patients with those mutations were favorable. In all cases, the tumor size was relatively small (1–3 cm) and there was no evidence of extrathyroidal invasion, lymph node or distant metastases. All patients were in remission after the initial management.

Apart from these rare non-hot spot *BRAF* mutations, *BRAF*^V600E^ was common in this series of adult DTCs. Similar to previous studies, we found a statistically significant association between the *BRAF*^V600E^ mutation and age (*P* < 0.0001), extrathyroidal invasion (*P* = 0.017), lymph node metastasis (*P* = 0.038) and TNM stage III/IV (*P* = 0.001) but not with other clinical and histopathological features such as gender, tumor size, tumor multifocality, distant metastasis, persistent and persistent/recurrent disease. This might be due to the relatively small sample size. To the best of our knowledge, this study is the first to determine the frequency of *BRAF* mutation exclusively in a pure sample of adult DTC patients. The previously reported studies were a mixture of adult and pediatric cases [19, 20]. *BRAF*^*V600E*^ mutation has been detected with a frequency ranging between ~30–85 % in classical PTC. On the other hand, in case of FVPTC, the frequency ranges between ~0 and 35 % [7, 11, 37]. The frequency of the *BRAF* mutations identified in our study is comparable to that of the other studies in the Middle East region [19, 20, 38].

## Conclusions

We have reported the frequency of exon 15 *BRAF* mutations (48.5 %) in a pure sample of adult DTC patients from an ethnically different population with a high rate of consanguinity and high rate of thyroid cancer. In addition to the common occurrence of *BRAF*^V600E^ mutation, we describe a relatively high rate of non-hotspot *BRAF* mutations (T599I, T599dup and K601E). These mutations were detected in 4 out of 204 cases (2 %) of DTC in our study. Our results show a high rate and a strong prognostic role of the classical *BRAF*^*V600E*^ mutation and also suggest a common occurrence of non-hot spot mutations in adult DTC from this highly inbred population.

## Abbreviations

DTC: differentiated thyroid cancer
PDTC: poorly differentiated thyroid cancer
CPTC: conventional papillary thyroid cancer
FVPTC: follicular variant thyroid cancer
TC-PTC: tall cell papillary thyroid cancer
HCC: hurthle cell carcinoma
DSC: diffuse sclerosing type papillary thyroid cancer
FTC: follicular thyroid cancer
FFPT: formalin fixed and paraffin embedded tissue
PCR: polymerase chain reaction
MAPK: mitogen activated protein kinase
PI3K: phosphatidylinositol-3-kinase

## References

[1] Davies L, Welch HG (2006). Increasing incidence of thyroid cancer in the United States, 1973–2002. JAMA.

[2] Jemal A, Bray F, Center MM, Ferlay J, Ward E, Forman D (2011). Global cancer statistics. CA Cancer J Clin.

[3] Pathology and genetics of tumours of endocrine organs. In: IARC WHO classification of tumours. World Health Organization; 2004.

[4] National Cancer Registry. Riyadh: 2007.

[5] Xing M (2013). Molecular pathogenesis and mechanisms of thyroid cancer. Nat Rev Cancer.

[6] Nikiforov YE, Nikiforova MN (2011). Molecular genetics and diagnosis of thyroid cancer. Nat Rev Endocrinol.

[7] Xing M (2005). BRAF mutation in thyroid cancer. Endocr Relat Cancer.

[8] Wan PT, Garnett MJ, Roe SM, Lee S, Niculescu-Duvaz D, Good VM, Jones CM, Marshall CJ, Springer CJ, Barford D, Marais R (2004). Mechanism of activation of the RAF-ERK signaling pathway by oncogenic mutations of B-RAF. Cell.

[9] Nikiforova MN, Kimura ET, Gandhi M, Biddinger PW, Knauf JA, Basolo F, Zhu Z, Giannini R, Salvatore G, Fusco A, Santoro M, Fagin JA, Nikiforov YE (2003). BRAF mutations in thyroid tumors are restricted to papillary carcinomas and anaplastic or poorly differentiated carcinomas arising from papillary carcinomas. J Clin Endocrinol Metab.

[10] Trovisco V, Soares P, Preto A, de Castro IV, Lima J, Castro P, Maximo V, Botelho T, Moreira S, Meireles AM, Magalhães J, Abrosimov A, Cameselle-Teijeiro J, Sobrinho-Simões M (2005). Type and prevalence of BRAF mutations are closely associated with papillary thyroid carcinoma histotype and patients’ age but not with tumour aggressiveness. Virchows Arch.

[11] Fugazzola L, Puxeddu E, Avenia N, Romei C, Cirello V, Cavaliere A, Faviana P, Mannavola D, Moretti S, Rossi S, Sculli M, Bottici V, Beck-Peccoz P, Pacini F, Pinchera A, Santeusanio F, Elisei R (2006). Correlation between B-RAFV600E mutation and clinico-pathologic parameters in papillary thyroid carcinoma: data from a multicentric Italian study and review of the literature. Endocr Relat Cancer.

[12] Eszlinger M, Niedziela M, Typlt E, Jaeschke H, Huth S, Schaarschmidt J, Aigner T, Trejster E, Krohn K, Bösenberg E, Paschke R (2014). Somatic mutations in 33 benign and malignant hot thyroid nodules in children and adolescents. Mol Cell Endocrinol.

[13] Alzahrani AS, Qasem E, Murugan AK, Al-Hindi HN, Alkhafaji DM, Almohanna M, Xing M, Alhomaidah D, Alswailem M (2015). Uncommon TERT promoter mutations in pediatric thyroid cancer. Thyroid.

[14] Alzahrani AS, Alkhafaji D, Tuli M, Al-Hindi H, Sadiq BB (2015). Comparison of differentiated thyroid cancer in children and adolescents (≤20 years) with young adults. Clin Endocrinol (Oxf).

[15] Kim SS, Kim SJ, Kim IJ, Kim BH, Jeon YK, Kim YK (2012). Comparison of clinical outcomes in differentiated thyroid carcinoma between children and young adult patients. Clin Nucl Med.

[16] Sassolas G, Hafdi-Nejjari Z, Casagranda L, Berger C, Bournaud C, Decaussin-Petrucci M, Berger N, Borson-Chazot F (2013). Thyroid cancers in children, adolescents, and young adults with and without a history of childhood exposure to therapeutic radiation for other cancers. Thyroid.

[17] El-Mouzan MI, Al-Salloum AA, Al-Herbish AS, Qurachi MM, Al-Omar AA (2007). Regional variations in the prevalence of consanguinity in Saudi Arabia. Saudi Med J.

[18] Kari JA, Bockenhauer D, Stanescu H, Gari M, Kleta R, Singh AK (2014). Consanguinity in Saudi Arabia: a unique opportunity for pediatric kidney research. Am J Kidney Dis.

[19] Abubaker J, Jehan Z, Bavi P, Sultana M, Al-Harbi S, Ibrahim M, Al-Nuaim A, Ahmed M, Amin T, Al-Fehaily M, Al-Sanea O, Al-Dayel F, Uddin S, Al-Kuraya KS (2008). Clinicopathological analysis of papillary thyroid cancer with PIK3CA alterations in a Middle Eastern population. J Clin Endocrinol Metab.

[20] Schulten HJ, Salama S, Al-Mansouri Z, Alotibi R, Al-Ghamdi K, Al-Hamour OA, Sayadi H, Al-Aradati H, Al-Johari A, Huwait E, Gari M, Al-Qahtani MH, Al-Maghrabi J, Sayadi H (2012). BRAF mutations in thyroid tumors from an ethnically diverse group. Hered Cancer Clin Pract.

[21] Murugan AK, Humudh EA, Qasem E, Al-Hindi H, Almohanna M, Hassan ZK, Alzahrani AS (2015). Absence of somatic mutations of the mTOR gene in differentiated thyroid cancer. Meta Gene.

[22] Cohen Y, Xing M, Mambo E, Guo Z, Wu G, Trink B, Beller U, Westra WH, Ladenson PW, Sidransky D (2003). BRAF mutation in papillary thyroid carcinoma. J Natl Cancer Inst.

[23] Givens DJ, Buchmann LO, Agarwal AM, Grimmer JF, Hunt JP (2014). BRAF V600E does not predict aggressive features of pediatric papillary thyroid carcinoma. Laryngoscope.

[24] Henke LE, Perkins SM, Pfeifer JD, Ma C, Chen Y, DeWees T, Grigsby PW (2014). BRAF V600E mutational status in pediatric thyroid cancer. Pediatr Blood Cancer.

[25] Francis GL, Waguespack SG, Bauer AJ, Angelos P, Benvenga S, Cerutti JM, Dinauer CA, Hamilton J, Hay ID, Luster M, Parisi MT, Rachmiel M, Thompson GB, Yamashita S (2015). The American thyroid association guidelines task force on pediatric thyroid cancer. Management guidelines for children with thyroid nodules and differentiated thyroid cancer. Thyroid.

[26] Rivera G, Lugo-Vicente H (2014). Thyroid cancer in children. Bol Asoc Med P R.

[27] Vaisman F, Corbo R, Vaisman M (2011). Thyroid carcinoma in children and adolescents-systematic review of the literature. J Thyroid Res.

[28] Wang JT, Huang R, Kuang AR (2014). Comparison of presentation and clinical outcome between children and young adults with differentiated thyroid cancer. Asian Pac J Cancer Prev.

[29] Matsuse M, Mitsutake N, Tanimura S, Ogi T, Nishihara E, Hirokawa M, Fuziwara CS, Saenko VA, Suzuki K, Miyauchi A, Yamashita S (2013). Functional characterization of the novel BRAF complex mutation, BRAF(V600delinsYM), identified in papillary thyroid carcinoma. Int J Cancer.

[30] De Falco V, Giannini R, Tamburrino A, Ugolini C, Lupi C, Puxeddu E, Santoro M, Basolo F (2008). Functional characterization of the novel T599I-VKSRdel BRAF mutation in a follicular variant papillary thyroid carcinoma. J Clin Endocrinol Metab.

[31] Chiosea S, Nikiforova M, Zuo H, Ogilvie J, Gandhi M, Seethala RR, Ohori NP, Nikiforov Y (2009). A novel complex BRAF mutation detected in a solid variant of papillary thyroid carcinoma. Endocr Pathol.

[32] Roskoski R (2010). RAF protein-serine/threonine kinases: structure and regulation. Biochem Biophys Res Commun.

[33] Eisenhardt AE, Olbrich H, Roring M, Janzarik W, Anh TN, Cin H, Remke M, Witt H, Korshunov A, Pfister SM, Omran H, Brummer T (2011). Functional characterization of a BRAF insertion mutant associated with pilocytic astrocytoma. Int J Cancer.

[34] Jones DT, Kocialkowski S, Liu L, Pearson DM, Ichimura K, Collins VP (2009). Oncogenic RAF1 rearrangement and a novel BRAF mutation as alternatives to KIAA1549:BRAF fusion in activating the MAPK pathway in pilocytic astrocytoma. Oncogene.

[35] Gauchotte G, Philippe C, Lacomme S, Leotard B, Wissler MP, Allou L, Toussaint B, Klein M, Vignaud JM, Bressenot A (2011). BRAF, p53 and SOX2 in anaplastic thyroid carcinoma: evidence for multistep carcinogenesis. Pathology.

[36] Afkhami M, Karunamurthy A, Chiosea S, Nikiforova MN, Seethala R, Nikiforov YE, Coyne C (2015). Histopathologic and clinical characterization of thyroid tumors carrying the BRAF mutation. Thyroid.

[37] Costa AM, Herrero A, Fresno MF, Heymann J, Alvarez JA, Cameselle-Teijeiro J, Garcia-Rostan G (2008). BRAF mutation associated with other genetic events identifies a subset of aggressive papillary thyroid carcinoma. Clin Endocrinol (Oxf).

[38] Frasca F, Nucera C, Pellegriti G, Gangemi P, Attard M, Stella M, Loda M, Vella V, Giordano C, Trimarchi F, Mazzon E, Belfiore A, Vigneri R (2008). BRAF(V600E) mutation and the biology of papillary thyroid cancer. Endocr Relat Cancer.

